# Effect of photobiomodulation therapy on neuronal injuries by ouabain: the regulation of Na, K-ATPase; Src; and mitogen-activated protein kinase signaling pathway

**DOI:** 10.1186/s12868-019-0499-3

**Published:** 2019-04-26

**Authors:** Yun-Hee Rhee, Jeong Hwan Moon, Jae Yun Jung, Connie Oh, Jin-Chul Ahn, Phil-Sang Chung

**Affiliations:** 1 Beckman Laser Institute Korea, Dankook University, Cheonan, 31116 Republic of Korea; 2 Laser Translational Clinical Trial Center, Dankook University Hospital, Cheonan, 31116 Republic of Korea; 3 Department of Otolaryngology-Head and Neck Surgery, College of Medicine, Dankook University, Cheonan, 31116 Republic of Korea; 4 Beckman Laser Institute and Medical Clinic, University of California, Irvine, 1002 Health Sciences Rd., Irvine, CA 92612 USA

**Keywords:** Photobiomodulation, Cortical neuron, Na, K-ATPase, Mitochondria membrane potential

## Abstract

**Background:**

To determine whether photobiomodulation (PBM) rescued the disruption of Na^+^/Ca^2+^ homeostasis and mitochondrial membrane potential by ouabain; the Na, K-ATPase inhibitor. For PBM in this study, a 660 nm LED array was used at energy densities of 0.78, 1.56, 3.12, 6.24, and 9.36 J/cm^2^.

**Results:**

HCN-2 neuronal cells treated with ouabain showed loss of cell polarity, disrupted cell morphology, and decreased cell viability, which were improved after PBM treatment. We found that ouabain-induced Na, K-ATPase inhibition promoted activation of downstream signaling through Src, Ras, and mitogen-activated protein kinase (MAPK), which were suppressed after PBM treatment. This provided evidence of Na, K-ATPase α-subunit inactivation and intracellular Ca^2+^ increase. In response to ouabain, we observed activation of Src and MAPK by Na, K-ATPase, decreased mitochondrial membrane potential, and Na^+^-dependent Ca^2+^ increases, which were restored by PBM treatment.

**Conclusions:**

This study demonstrated that Na^+^/K^+^ imbalance could be regulated by PBM treatment in neuronal cells, and we suggest that PBM is a potential therapeutic tool for Na, K-ATPase targeted neuronal diseases.

**Electronic supplementary material:**

The online version of this article (10.1186/s12868-019-0499-3) contains supplementary material, which is available to authorized users.

## Background

Neuronal activity can be manipulated through molecular mechanisms at several levels: (1) ion channels, (2) neurotransmitters and their receptors, (3) auxiliary intramembranous or cytoplasmic signal transducing molecules, and (4) neurotransmitter transporters. These molecular mechanisms facilitate their conservation through reaccumulation in the terminal and then synaptic vesicles of these molecular entities such as neurotransmitters and neurotransmitter transporters to regulate three major cations; Na^+^, K^+^, and Ca^2+^ [1–3]. The balance of these major cations has a crucial role in neuronal activity and is maintained by Na, K-ATPase. The Na, K-ATPase is a plasma membrane protein complex which activates the ion transport system to generate Na^+^ and K^+^ gradients across the cell plasma membrane [2, 4], and mediate the effects of endogenous digitalis-like compounds such as ouabain in the cell [5]. The Na, K-ATPase is composed of catalytic α and glycosylated β subunit [6]. Especially, the activity of α subunit in Na, K-ATPase is inhibited by ouabain binding [7]. Ouabain is well-known to prolong depolarization of neurons leading to osmolysis or calcium necrosis in brain tissues [8]. Upon ouabain binding, the Na, K-ATPase initiates a series of reactions that include interaction with neighboring proteins in what has been described as the Na, K-ATPase signal [9, 10]. In our previous study, we suggested that photobiomodulation (PBM) by low-level laser therapy had the potential to rescue auditory neuropathy induced by ouabain [11]. PBM has been used in a variety of applications, such as wound healing [12], inflammation [13], pain relief [14], and tissue regeneration [15]. Although physiological improvement following PBM therapy has been reported, studies investigating the molecular mechanism remain few. In the present study, we provide the evidence that protective effect of PBM on ouabin-induced Na, K-ATPase disruption through Src/Ras/MAPK in neuronal cells.

## Methods

### Cells

The human brain cortical neuron cell line HCN-2 (ATCC CRL-10742) was purchased from ATCC (Manassas, VA, USA) and was maintained in Dulbecco’s Modified Eagle Media (DMEM) supplemented with 4 mM l-glutamine, 4.5 g/L glucose, and 10% fetal bovine serum, which were purchased from Life Technologies (Grand Island, NY, USA).

### Chemicals and antibodies

Ouabain, 3-(4,5-dimethyl-thiazol-2-yl)-2,5-diphenyltetrazolium (MTT), tetramethylrhodamine ester (TMRE), and β-actin were purchased from Sigma Aldrich (St. Louis, MO, USA). Phospho-Na, K-ATPase α; Na, K-ATPase α; phospho-SRC; and RAS were purchased from Abcam (Cambridge, MA, USA). Phospho-ERK, ERK, phospho-JNK, JNK, phospho-p38, and p38 were purchased from Cell Signaling (Beverly, MA, USA). Anti-mouse or anti-rabbit HRP-conjugated IgG antibodies were purchased from Santa Cruz (Santa Cruz, CA, USA) (Additional files 1, 2).

### PBM conditions by low-level light

**Fig. 1:**
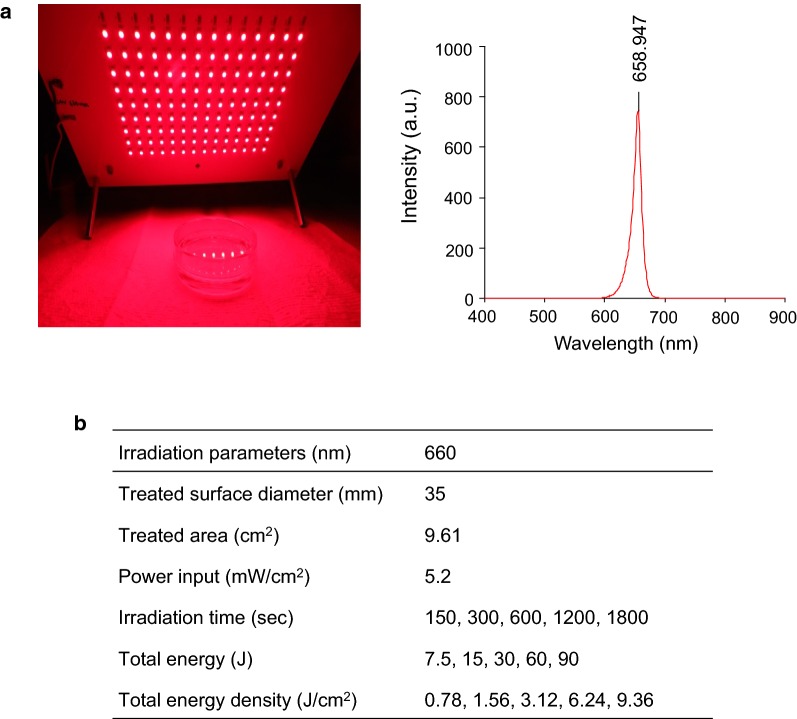
The figure of light emitting diode. The light source was a continuous wave (CW) type 660 mM emitting diode, which was manufactured by WON Technology (**a**). Total energy was modulated with different time intervals (**b**)

The light source was a continuous wave (CW) type of 660 nm light emitting diode, which was manufactured by WON Technology Co., Ltd., Korea. Total energy was modulated with different time intervals, and the power input was fixed at 50 mW. The irradiance at the surface of the cell monolayer was measured with a power meter (Orion, Ophir Optronics Ltd., UT, USA). The LED panel and wavelength are shown in Fig. 1a, and the condition of PBM treatment is described in Fig. 1b.

### Cell viability assay

Cells were cultured at a density of 5000 cells/well in 96-well plates at 5% CO_2_ and 37 °C. After 24 h, cells were exposed to various concentrations of ouabain and were irradiated by 660 nm LED at 50 mW 30 min later. The irradiated cells were incubated for 2 or 24 h, and then MTT was added at a final concentration of 0.5 mg/mL. After 2 h of incubation, the reaction was stopped by adding a lysis solution (20% SDS, 50% dimethylformamide). The relative optical density for each well was determined at 450 nm by a microplate spectrophotometer (Bio-Tek, Winooski, VT, USA). Cell viability was calculated as a percentage of the ouabain-treated group versus the untreated control group.

### Cell morphological observation

We modulated the ouabain exposure time for the optimal time point of cell survival by PBM. Cells were exposed to 5 mM ouabain and PBM treatment followed after 30 min. Cells were incubated for 2, 4, 8, or 24 h with ouabain, and cell morphological changes were observed using an inverted microscope (Olympus CKX53, Miami, FL, USA).

### Na/K-ATPase activity assay

Cells were treated with ouabain at the same concentration (0, 3, 5, 10, 30 mM) and exposure conditions as in cell viability assay and Na/K-ATPase activity with or without PBM was measured using the Na/K-ATPase activity assay kit according to manufacturer (Mybioscience, SanDiego, CA, USA). Briefly, cells were lysed with rapid freezing and thawing with dry ice and acetone, and assayed for Na/K-ATPase activity. The Na^+^/K^+^-ATPase activity was measured using an end-point phosphate ATP hydrolysis protocol performed. The inorganic phosphate released from the cells was measured using colourimetric assays and expressed in μmol per mg protein.

### Intracellular ADP/ATP ratio assay

ADP/ATP ratio with or without PBM under same condition of ouabain was also measured using the ADP/ATP ratio assay kit according to manufacturer (Abcam, Cambridge, MA, USA). Briefly, cells were incubated with nucleotide releasing buffer for 5 min after various concentrations of ouabain exposure. 100 μL ATP monitoring enzyme and nucleotide releasing mix was added onto lunimoscence plate, and background values (A) were measured using luminometer (Synergy/MTX, BioTek Instruments, Winoosk, VT, USA). 50 μL of samples were added onto ATP monitoring enzyme contained plate, then the values (B) were measured after 2 min. The ADP level was also measured before (C) and after (D) of adding ADP converting enzyme as same method as described previously. The ADP/ATP ration was calculated as follows: ADP/ATP ratio = (B − A)/(D − C).

### Western blotting analysis

Cells were lysed in protein lysis buffer (25 mM Tris–HCl (pH 7.4), 150 mM NaCl, 5 mM EDTA, 0.1% SDS) containing protease and phosphatase inhibitors (Sigma Aldrich). Protein concentration was determined using the BCA assay (Bio-Rad Laboratories, Hercules, CA, USA). Protein samples (30 μg) were separated by SDS–polyacrylamide gel electrophoresis (SDS-PAGE) and transferred onto a PVDF membrane (Bio-Rad Laboratories). The blotting membrane was incubated with primary antibodies overnight: phospho-Na, K-ATPase α; phospho-SRC; phospho-ERK; phospho-JNK; phospho-p38 (1:500); Na, K-ATPase α; RAS; ERK; JNK; p38 (1:1000); and β-actin (1:5000). The blots were incubated for 2 h at room temperature with secondary HRP-conjugated antibodies (1:2000). The signals were quantified and analyzed using the NIH imaging software, Image J (NIH, Bethesda, MD, USA). The level of protein expression was normalized to β-actin. The value of protein levels was designated as one in the control group. The results were expressed as the mean proportion of the control group values.

### Cellular calcium ion measurements

Intracellular free Ca^2+^ was measured using the Fluo-8-no wash calcium assay kit (Abcam). Fluo-8 epifluorescence was excited at 490 nm and images were obtained at 520 nm. The imaging data were collected with a fluorescence microscopy system (Olympus BX51), and the intensity of fluorescence was determined with a fluorometer (Bio-Tek Instruments).

### Mitochondrial membrane potential measurements

Cells were treated with ouabain and then loaded immediately with 200 nM TMRE (Invitrogen, Eugene, OR, USA) for 30 min in the dark. Cells were subjected to the same treatment as described above prior to imaging. The observation and intensity measurement was performed with a confocal microscope (LSM510, Carl Zeiss, Switzerland).

### Statistical analysis

The results are expressed as the mean ± SD. The values of cell viability, western blot anaylsis, calcium ion measurement, mitochondrial membrane potential measurement were compared using one-way ANOVA (Tukey test). The cell viability, Na,K-ATPase activity, and ADP/ATP ratio analysis were compared using two-way ANOVA (Bonferroni post-test). All data were analyzed using Graph Pad, Prism^®^ (La Jolla, CA, USA). Statistical significance shows *p < 0.1, **p < 0.05, and ***p < 0.001.

## Results

### Ouabain-induced cytotoxicity in HCN-2 cells

**Fig. 2:**
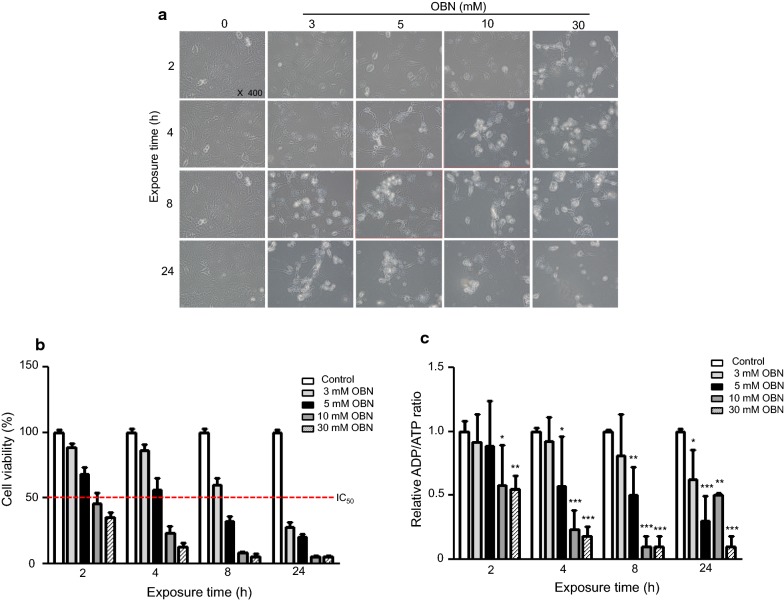
Cells were exposed to various concentrations of ouabain and incubated for 2, 4, 8, or 24 h. The morphological changes of HCN-2 cells were observed using an inverted microscope and photographed at 200 × magnification (**a**). The viability of HCN-2 cells treated with ouabain was measured by MTT assay. Survival rate analysis from the various concentrations (3, 5, 10, and 30 mM) and exposure times (2, 4, 8, and 24 h) of ouabain revealed the ideal toxic conditions for HCN-2 cells to be a 4 h exposure with 5 mM ouabain. Every assay was performed three times, and the results are expressed as the mean ± SD (**b**)

To determine the optimal concentration and exposure time of ouabain to HCN-2 neuronal cells, the 3-(4,5-dimethyl-thiazol-2-yl)-2,5-diphenyltetrazolium (MTT) assay was performed with various treatment schemes. Cells were treated with ouabain at a concentration of 3, 5, 10, or 30 mM and were then subjected to incubation for 2, 4, 8, or 24 h. Fresh normal media was changed at the end of the exposure time. The MTT assay was performed 24 h after exchanging the media. Ouabain decreased cell viability in both a time- and dose-dependent manner. Increasing the ouabain concentration from 3 to 30 mM significantly augmented the cytotoxic effect at all time points. As shown in Fig. 2a, cells were floating or exhibited altered morphology after 8 h with 5 mM of ouabain, and cell apoptosis occurred within 4 h with 10 mM of ouabain. The survival rate according to the concentration of ouabain and exposure time is depicted in Fig. 2b. Through the analysis of survival rates following ouabain treatment, we determined the ideal toxic condition for HCN-2 cells to be a 4 h exposure to 5 mM ouabain.

### PBM effect on cell viability

To address whether PBM could promote cell survival under cytotoxic conditions in HCN-2 cells, we treated cells with low-level light irradiation (LLLI). Ouabain-treated HCN-2 cells were exposed to LLLI with various energy densities ranging from 0.78 to 9.36 J/cm^2^. The power of the LLLI was fixed at 50 mW and total energy density was modulated by time of irradiation. We subscribed to the total energy density and duration time presented in Fig. 1b.

**Fig. 3:**
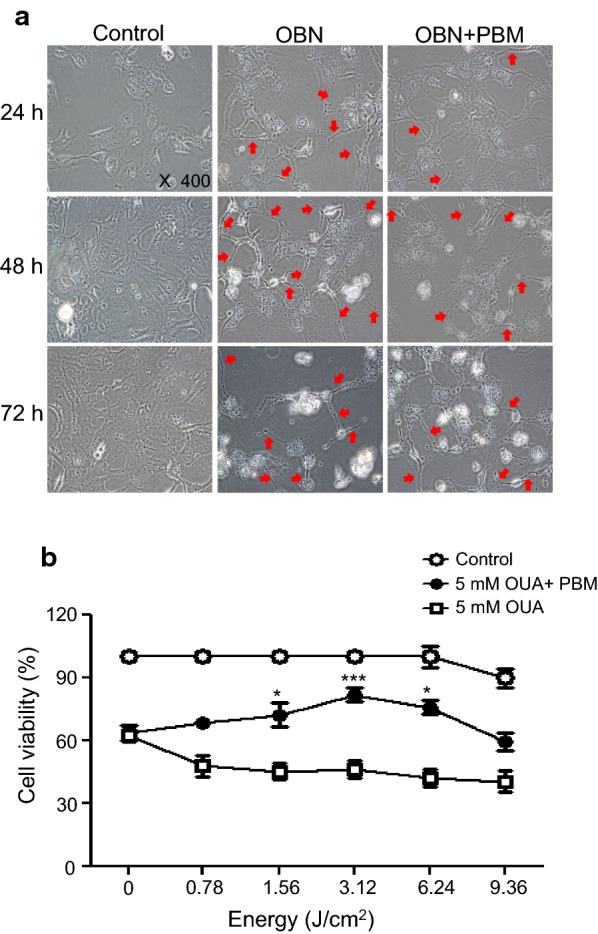
Irradiation was performed 30 min after 5 mM ouabain treatment. Cells were incubated for 24, 48, or 72 h with ouabain, and cell morphological changes were observed using an inverted microscope and photographed at 200 × magnification (**a**). The viability of HCN-2 cells with ouabain after irradiation with various energy doses was measured by MTT assay. Cell survival increased from 64.7 to 81.8% at 3.12 J/cm^2^ irradiation. However, there was a decrease in cell viability at 9.36 J/cm^2^ irradiation (**b**). Every assay was performed three times, and the results are expressed as the mean ± SD. *p < 0.1 and ***p < 0.001

Cell viability and shape were then observed from 24 to 72 h. As shown in Fig. 3, ouabain exposed cells were deformed and the survival rate was decreased to less than 30% at 72 h. However, the survival rate of ouabain exposed cells was significantly elevated after LLLI. Cell survival with 5 mM ouabain was 45.1% before PBM, however it was increased to 68.59 ± 2.88% with 0.78 J/cm^2^ irradiation (*p* < 0.001, t = 9.304), 72.39 ± 5.46% with 1.56 J/cm^2^ irradiation (*p* < 0.001, t = 12.33), 81.81 ± 3.39% with 3.12 J/cm^2^ irradiation (*p* < 0.001, t = 16.12), 75.94 ± 3.41% with 6.24 J/cm^2^ irradiation (*p* < 0.001, t = 15.29), and 59.67 ± 4.13% with 9.36 J/cm^2^ irradiation (*p* < 0.001, t = 8.652). However, there was a decrease in cell viability in the 9.36 J/cm^2^ irradiation group (q = 2.88). When comparing only ouabain with PBM treated group, cell survival was significantly increased 1.56 J/cm^2^ (q = 4.76), 3.12 J/cm^2^ (q = 7.84), and 6.24 J/cm^2^ (q = 4.82) irradiation group. These results suggest that PBM was effective against cell damage at a specific energy dose, not energy does-dependent.

### The effect of PBM on ouabain-induced Na, K-ATPase activity

**Fig. 4:**
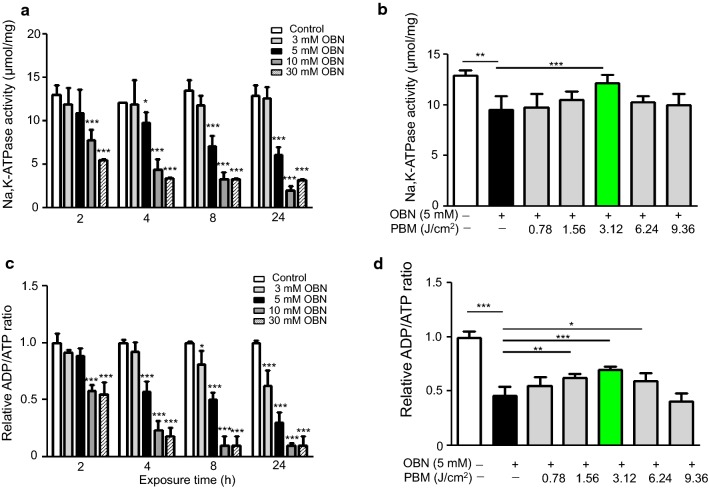
Cells were treated with ouabain at the same concentration and exposure conditions as in cell viability assay and Na/K-ATPase activity with or without PBM was measured using the Na/K-ATPase activity assay kit. Ouabain inhibited Na/K-ATPase activity more 5 mM of concentration and 4 h exposure (**a**). In contrast, Na/K-ATPase activity was increased after PBM (**b**). ADP/ATP ratio were calculated the division of ADP converting enzyme differences per ATP monitoring enzyme values. ADP/ATP ratio was decreased at 3 mM ouabain for 8 h and at 5 mM ouabain for 4 h. Over 10 mM of ouabain, no value was measured (**c**). However, the ratio of ADP/ATP was increased after PBM with energy dose except the highest dose at 9.36 J/cm^2^ (**d**). Every assay was performed five times, and the results are expressed as the mean ± SD. *p < 0.1, **p < 0.05, and ***p < 0.001

Ouabain is known to induce Na +/K + imbalance through Na, K-ATPase inhibition [16]. We evaluated the Na, K-ATPase activity using inorganic phosphate colorimeric assay. Figure 4a showed the Na, K-ATPase activity of HCN-2 cells according to concentration of ouabain. 3 mM ouabain treatment had no effect to Na/K-ATPase activity. After 5 mM ouabain treatment, Na, K-ATPase activity was decreased to 9.81 ± 1.19 μmol/mg (*p* < 0.05, t = 2.646) at 4 h, 7.13 ± 1.16 μmol/mg (*p* < 0.001, t = 6.921) at 8 h, and 6.1 ± 0.95 μmol/mg (*p* < 0.001, t = 7.368) at 24 h. From 10 mM or more, Na/K-ATPase activity was decreased as the blank control. However, Na/K-ATPase activity was recovered from 9.5 ± 1.34 μmol/mg (*p* < 0.001, q = 7.67) to 12.18 ± 0.78 μmol/mg with 3.12 J/cm^2^ irradiation (*p* < 0.05, q = 5.971). There were no significances differentiation other irradiation groups (Fig. 4b).

### The effect of PBM on ouabain-induced intracellular ATP/ADP ratio

Ouabain is also known to induce Na +/K + imbalance and result in the accumulation of Na^+^ ions in the cells, which induce intracellular stress. ATP is an energy source of NA^+^/K^+^ transport activation which is inhibited specifically by ouabain. In order to determine the extent of ATP, we assessed intracellular ADP/ATP ratio using luminescence assay. Figure 4c showed the ADP/ATP ratio of HCN-2 cells according to concentration and time after ouabain treatment. The ADP/ATP ratio was decreased to 0.81 ± 0.12 (*p* < 0.05, t = 3.106) at 8 h and to 0.62 ± 0.14 (*p* < 0.001, t = 6.262) at 24 h after 3 mM ouabain treatment. After 5 mM ouabain treatment, the ADP/ATP ratio decreased to 0.53 ± 0.09 (*p* < 0.001, t = 7.126) at 4 h, 0.53 ± 0.06 (*p* < 0.001, t = 8.255) at 8 h, and 0.30 ± 0.09 (*p* < 0.001, t = 11.56) at 24 h. From 10 mM or more, it decreased to less than 50% after 2 h, and no measured value was observed. To determine whether PBM restores the accumulation of ATP by ouabain, we evaluated the ADP/ATP ratio after LLLI. The ratio of ADP/ATP was elevated from 0.47 ± 0.08 (*p* < 0.001, q = 18.82) to 0.54 ± 0.08 with 1.56 J/cm^2^ irradiation (*p* < 0.05, q = 5.63), 0.70 ± 0.03 with 3.12 J/cm^2^ irradiation (*p* < 0.001, q = 8.25), 0.59 ± 0.07 with 6.24 J/cm^2^ irradiation (*p* < 0.1, q = 4.67). There was no significance differentiation both 0.78 J/cm^2^ and 9.36 J/cm^2^ irradiation group (Fig. 4d). These results also support that PBM was effective against cell damage at a specific energy dose, not energy does-dependent.

### The effect of PBM on ouabain-induced Na, K-ATPase cascade in HCN-2 cells

**Fig. 5:**
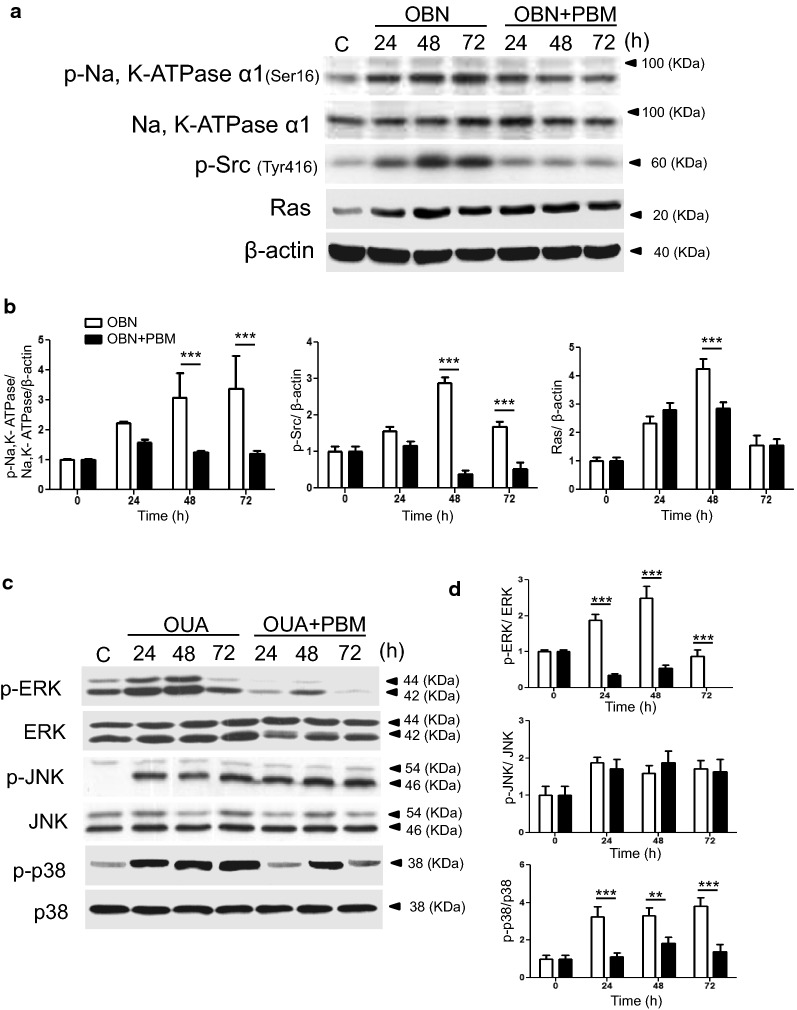
The levels of Na, K-ATPase α; Src; Ras (**a**); phospho-ERK/ERK; phospho-JNK/JNK; and phospho-p38/p38 (**c**) were analyzed by western blot. The expression of phospho-Na, K-ATPase; phospho-Src; Ras; and MAPK were increased by ouabain, but decreased after PBM treatment. The level of protein expression was normalized to β-actin and the signal quantifications were analyzed with the NIH imaging software, Image J ^®^. The results are expressed as the mean ± SD. *p < 0.1, **p < 0.05, ***p < 0.001 (**b**, **d**)

It was suggested that Na, K-ATPase inhibition by ouabain had been linked to the Src-Ras-p42/44 MAPK cascade [17]. Especially, Na, K-ATPase phosphorylation by ouabain was involved with the activity of Src [16]. We determined whether PBM reduced the intracellular stress by ouabain through Na, K-ATPase-Src-Ras pathway using western blot analysis. Figure 5a showed the expression of phospho-Na, K-ATPase; phospho-Src; and Ras, Ouabain increased the phosphorylation of Na, K-ATPase up to 2.2 folds at 24 h maintained to 72 h. Ouabain also increased the phosphorylation of Src up to 2.89 folds at 48 h. The expression of Ras was increased up to 4.26 folds at 48 h by ouabain. However, PBM reduced the expression of Src and Ras as well as Na, K-ATPase. The phosphorylation of Na, K-ATPase by ouabain was decreased more than 50% after PBM (*p* < 0.001, F = 19.62). The level of p-Src and Ras was also decreased more than 25% (*p* < 0.001, F = 4.54) and 50% (*p* < 0.001, F = 2.35) after PBM (Additional file 3).

### The effect of PBM on ouabain-induced MAPK signaling in HCN-2 cells

Next, we measured the level of phosphorylation of MAPK by ouabain with or without PBM. As shown in Fig. 5c, the phosphorylation of ERK began to increase at 24 h (1.89 folds), reached a maximum at 48 h (2.5 folds), and decreased at 72 h (0.9 folds) by ouabain. The phosphorylation of p38 and JNK also began to increase at 24 h (3.2 and 1.8 folds) and maintained to 72 h (3.8 and 1.7 folds) by ouabain. These increase of MAPK by ouabain was significantly inhibited by PBM treatment (*p* < 0.001, F_ERK_ = 3.56, F_p38_ = 11.05) except JNK (Additional file 4).

### The effect of PBM on ouabain-induced intracellular Ca^2+^ levels in HCN-2 cells

**Fig. 6:**
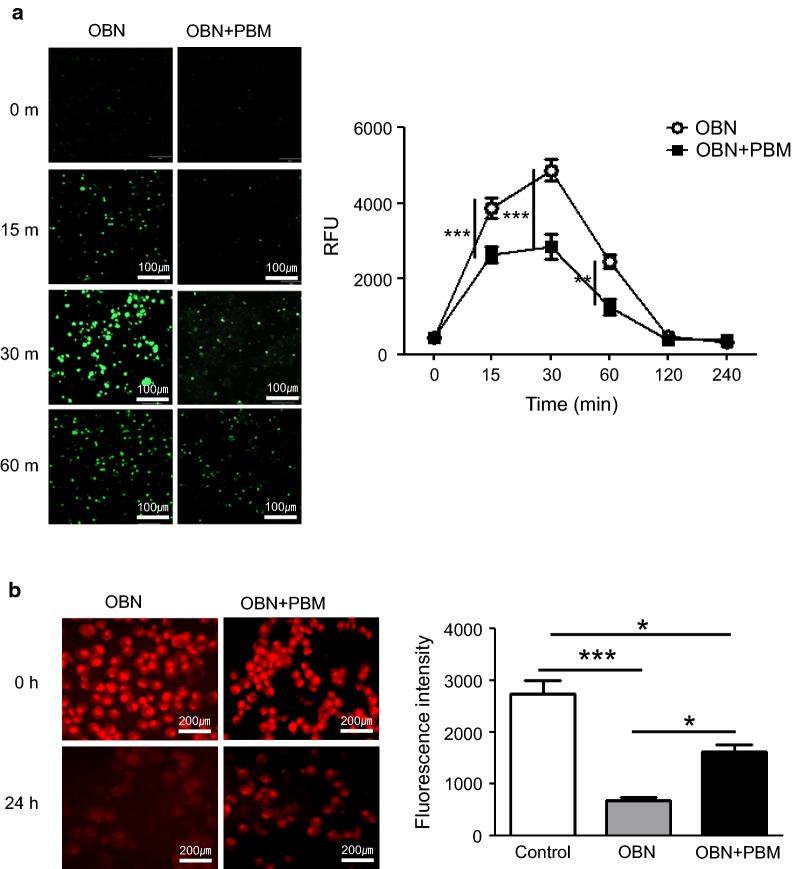
Ouabain increased intracellular Ca^+^ as early as 15 min after addition, and the effect lasted for up to 60 min, which was negatively regulated by PBM (**a**). Ouabain caused a marked decrease in orange to red fluorescence of TMRE, indicating a significant loss of mitochondrial membrane potential and damage to the cells (**b**). The results are expressed as the mean ± SD. *p < 0.1, **p < 0.05, ***p < 0.001

It has been known that cell necrosis is induced by accumulation of intracellular Na^+^ by blocking the Na^+^/K^+^ pump and Ca^2+^ [2, 9]. Thus, we measured intracellular Ca^2+^ using the cell permeable indicator Fluo-8-AM. As shown in Fig. 6a, ouabain increased intracellular Ca^+^ as early as 15 min after addition, and the effect lasted for up to 60 min, which was regulated by PBM. We also measured the intracellular Ca^2+^ concentration. The values of intracellular Ca^2+^ were 3881 ± 261.5 at 15 min, 4869 ± 288.1 at 30 min, and 1969 ± 186.4 at 60 min, and which were reduced to 2649 ± 216.3 at 15 min (*p* < 0.001, t = 10.42), 2849 ± 322.1 at 30 min (*p* < 0.001, t = 17.09), and 1260 ± 217.5 at 60 min (*p* < 0.05, t = 5.15). This result suggested that PBM effectively prevented the Ca^2+^ increase during ouabain-induced cytotoxicity.

### The effect of PBM on ouabain-induced mitochondrial membrane potential in HCN-2 cells

The mitochondrial membrane potential seems to be very important to forms the transmembrane potential of hydrogen ions [18]. Whether PBM could restore the disrupted mitochondrial membrane potential by ouabain, we determined changes in mitochondrial membrane potential [19, 20]. Figure 6b showed that ouabain cause marked decrease in red fluorescence of TMRE, which indicating a significant loss of mitochondrial membrane potential of cells (*p* < 0.001, q = 11.43). The reduced mitochondrial membrane potential was reversed after PBM treatment (*p* < 0.1, q = 5.29) which suggested that PBM effectively protected the mitochondrial membrane potential disruption by ouabain.

## Discussion

The mechanisms of cell recovery induced by PBM in injured cells are not well defined. All photobiological responses are determined by the absorption of energy by photoacceptor molecules during light irradiation. Photon absorption converts light into signals that can stimulate biological processes. The near-infrared (NIR) light could modulate signaling pathways and regulate reactive oxygen species (ROS), adenosine tri-phosphate (ATP), Ca^2+^, and NO, which affect cell homeostasis, cytoskeleton reorganization, and cell proliferation/differentiation [21–24]. Although the biochemical and pharmacological properties of the Na, K-ATPase have been studied in various cell lines, the study of PBM effect on Na, K-ATPase has not been reported. In previous study, we observed that PBM rescued auditory neuropathy induced by ouabain in vivo, and these results led us to explore the further study [11]. Thus in this study, we investigated the signaling pathways responsible for mediating the effects of PBM using HCN-2 human cortical neuronal cell line which was well defined in terms of neurotoxicity and a model for Alzheimer’s disease [25]. As an initial test for ouabain-induced cytotoxicity in human neuronal cells, we exposed HCN-2 cells to various concentrations of ouabain and observed the cell morphological changes with or without PBM. It is important to observe cell morphological changes because the ouabain-induced inhibition of Na, K-ATPase reduces cellular polarization (depolarizing effect). We elucidated that a sub-lethal dose of ouabain for HCN-2 cells was defined as 5 mM for 4 h through morphological observation and Na, K-ATPase activity analysis. To determine ATP accumulation by NA, K-ATPase inhibition by ouabain in HCN-2 cells, the ADP/ATP ratio assay was performed. We observed that ATP accumulation began at 3 mM of ouabain for 8 h and 5 mM of ouabain for 4 h, which were the same as the cell viability analysis and morphology observation. As previously described, we have also observed degeneration of sparing the sensory neural cells that were exposed to 3 mM ouabain for 1 h in animal study [11]. Importantly, PBM increased the cell viability and the ADP/ATP ratio with energy density except at the highest density of 9.36 J/cm^2^ (Figs. 3, 4). Consistent with these results, transcranial LLLI for traumatic brain injury in mice showed biphasic neurological effects and energy density for treatment was varied [26]. These results suggested that PBM had an effective energy dose against cell damage (Additional file 5).

Na, K-ATPase is composed of three subunits: α-subunit, β-subunit, and γ-subunit, and functions in cellular electrochemical gradient maintenance, osmotic balance, cell adhesion and motility, and initiation of intracellular signaling [27–29]. We focused on the α-subunit because the binding sites for ATP, Mg^2+^, and cardiac glycoside, as well as Na^+^ and K^+^ ions, are all located in the α-subunit [2, 9, 10]. Na, K-ATPase induced energy deficiency and dysfunction are common consequences of many pathological insults [2, 29, 30], and multiple cell signaling pathways response to digitalis drugs such as ouabain [31, 32]. Moreover, it is known that Src bind to α-subunit of Na, K-ATPase in an inactivated state and that is activated with disruption of α-subunit by ouabain [31, 33]. It has been also reported that Na, K-ATPase phosphorylation by ouabain activated the kinase Src and downstream members of the MAPK pathway [34]. To determine whether PBM regulates the Na, K-ATPase phosphorylation and downstream members such as Src, Ras and MAPK, we performed western blot analysis. Western blot analysis revealed that Na, K-ATPase activity was inhibited by ouabain; however, which was restored by PBM. In addition, PBM regulated the activation of Src, Ras and MAPK except JNK (Fig. 5). Src activation is dependent on concentrations of other specific ligands of Na, K-ATPase: Na^+^, K^+^, vanadate, ATP, and ADP [35]. Although Src activation has known to be involved in many signaling cascade, the result of intracellular ADP/ATP ratio with ouabain (Fig. 4) revealed that Src activation was related to the up regulation of ATP by NA, K-ATPase inhibition, and this process could be controlled by PBM. Our results showed that for the first time PBM modulated the activation of Src and MAPK by restoring the NA, K-ATPase and intracellular ADP/ATP ratio (Additional file 6).

Meanwhile, blocking Na, K-ATPase has two direct and marked impacts on cellular ionic homeostasis: increased intracellular Na^+^ concentration and decreased intracellular K^+^ concentration [36]. PBM is known to regulate ATP and Ca^2+^ release, which could contribute to the regulation of Na, K-ATPase and its downstream pathway. Inhibition of Na, K-ATPase raises the intracellular Na^+^ concentration and increases the intracellular Ca^2+^ concentration via the sodium-calcium exchanger [1, 5, 37]. Thus, we investigated the level of intracellular Ca^+^ using the Fluo-8-AM indicator. We observed that Ca^+^ was increased within 15 min of ouabain treatment and was maintained to 30 min. However, PBM treatment suppressed the accumulation of intracellular Ca^+^ levels, and several additional reports support this finding. Sassoli et al. [38] reported that low intensity 635 nm diode laser irradiation inhibited fibroblast transition through transient receptor potential channel expression, and de Freitas and Hamblin proposed a mechanism of PBM that activated ROS, cyclic adenosine monophosphate (cAMP), NO, and Ca^2+^, leading to activation of transcription factors [22].

The inhibition of Na, K-ATPase by ouabain is also well-known to be cytotoxic to a variety of normal cells through decreased mitochondrial membrane potential and caspase activation [39]. We observed mitochondrial membrane potential using TMRE staining and found that PBM treatment could rescue the mitochondrial membrane potential degeneration by ouabain. Apoptotic features has been known to include phosphatidylserine translocation, caspase activation, Ca^2+^ increase, and the disruption of the plasma membrane which were due to MAPK activation through a Na, K-ATPase/Src/Ras signaling [2, 37]. Our present study suggested that PBM blocked the elevation of Na, K-ATPase/Src/Ras signaling and the decrease of mitochondrial membrane potential.

## Conclusion

PBM inhibited the decline of ouabain-induced cell viability, and this result was due to modulate of Na, K-ATPase α-subunit and intracellular Ca^2+^ increase. The activation of Na, K-ATPase, Src, and MAPK and the decrease of mitochondrial membrane potential by ouabain were restored by PBM. In conclusion, we suggest that neuronal cell recovery by PBM through Na, K-ATPase/Src/MAPK regulation is a potential therapeutic tool for Na, K-ATPase targeted neuronal diseases.

## Additional files

**Additional file 1.** The raw data of western blot. NaK-ATPase, ERK, and JNK in Fig. 5a, c.

**Additional file 2.** The raw data of western blot. p-NaK-ATPase and p-p38 in Fig. 5a, c.

**Additional file 3.** The raw data of western blot. p-SRC and β-actin in Fig. 5a, c.

**Additional file 4.** The raw data of western blot. Ras, ERK, and p38 in Fig. 5a, c.

**Additional file 5.** The raw data of Na, K-ATPase activity analysis in Fig. 4a, b.

**Additional file 6.** The raw data of ADP/ATP ratio analysis in Fig. 4c, d.

## Abbreviations

PBM: photobiomodulatoin
MAPK: mitogen-activated protein kinase
DMEM: Dulbecco’s Modified Eagle Media
MTT: 3-(4,5-dimethyl-thiazol-2-yl)-2,5-diphenyltetrazolium
CW: continuous wave
LLLI: low-level light irradiation
ERK: extracellular-signal-regulated kinase
JNK: c-Jun N-terminal kinase
TMRE: tetramethylrhodamine ester
NIR: near-infrared
ROS: reactive oxygen species
ATP: adenosine tri-phosphate
NO: nitric oxide
cAMP: cyclic adenosine monophosphate
