# Supplementary figures and images for: Effect of photobiomodulation therapy on neuronal injuries by ouabain: the regulation of Na, K-ATPase; Src; and mitogen-activated protein kinase signaling pathway
Source: BMC Neurosci. 2019 Apr 26;20:19. doi: 10.1186/s12868-019-0499-3 (PMC6486688; doi:10.1186/s12868-019-0499-3)

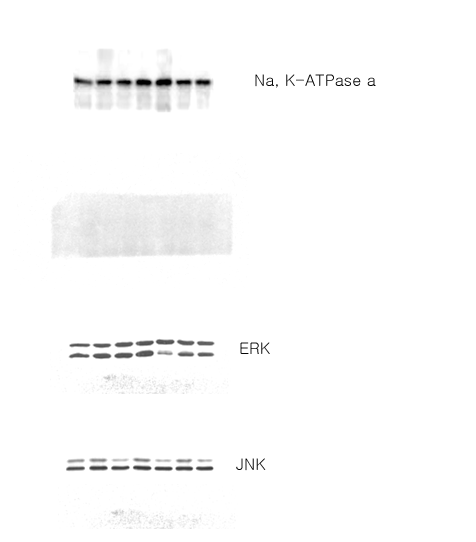

Supplement: Supplementary file 1 — Additional file 1. The raw data of western blot. NaK-ATPase, ERK, and JNK in Fig. 5a, c. [file 12868_2019_499_MOESM1_ESM.tif]

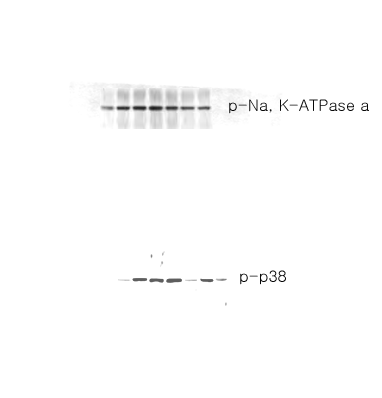

Supplement: Supplementary file 2 — Additional file 2. The raw data of western blot. p-NaK-ATPase and p-p38 in Fig. 5a, c. [file 12868_2019_499_MOESM2_ESM.tif]

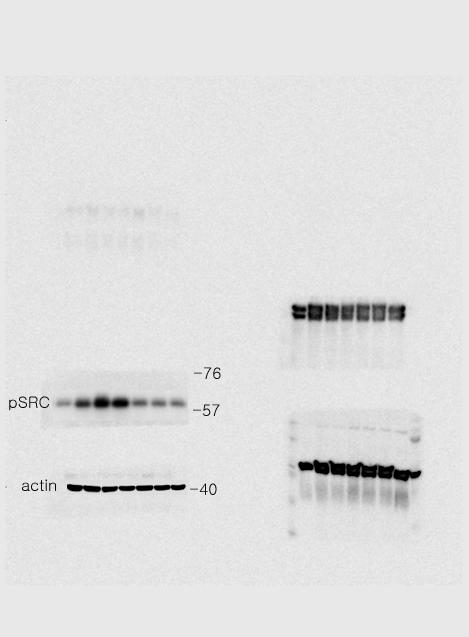

Supplement: Supplementary file 3 — Additional file 3. The raw data of western blot. p-SRC and β-actin in Fig. 5a, c. [file 12868_2019_499_MOESM3_ESM.tif]

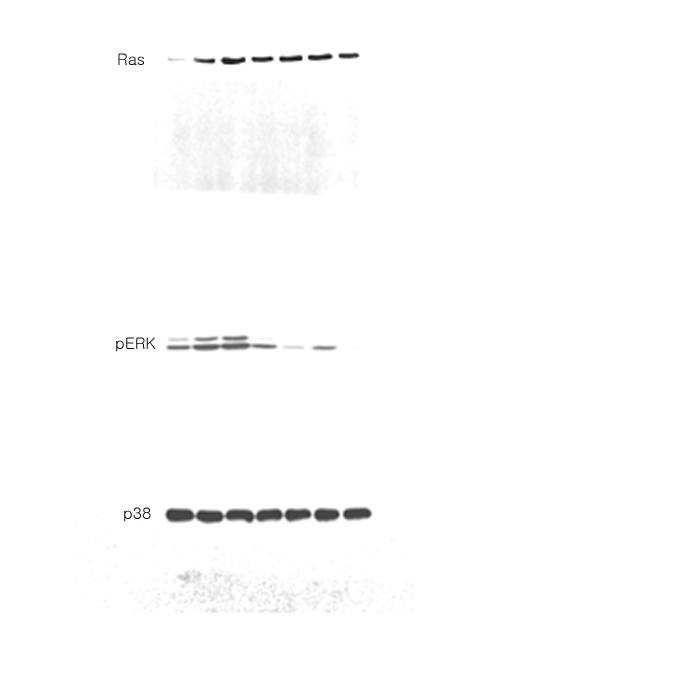

Supplement: Supplementary file 4 — Additional file 4. The raw data of western blot. Ras, ERK, and p38 in Fig. 5a, c. [file 12868_2019_499_MOESM4_ESM.tif]
